# Trimethylamine N-oxide (TMAO) in heat stroke: molecular mechanisms from gut dysbiosis to multi-organ injury

**DOI:** 10.3389/fphys.2026.1983362

**Published:** 2026-10-23

**Authors:** Qiong Wu, Guodong Lin, Yuanyuan Ou, Bo Ji, Zhiguo Pan, Yu Shao

**Affiliations:** 1 School of Pharmacy, Southern Medical University, Guangzhou, China; 2 Department of Pharmacy, People's Liberation Army General Hospital of Southern Theater Command, Guangzhou, China; 3 The First School of Clinical Medicine, Southern Medical University, Guangzhou, China; 4 The First School of Clinical Medicine, Guangzhou University of Chinese Medicine, Guangzhou, China; 5 Department of Emergency, People's Liberation Army General Hospital of Southern Theater Command, Guangzhou, China; 6 Department of General Medicine, People's Liberation Army General Hospital of Southern Theater Command, Guangzhou, China

**Keywords:** endothelial dysfunction, gut microbiota, heat stroke, mitochondrial dysfunction, NLRP3 inflammasome, thrombosis, trimethylamine n-oxide

## Abstract

Heat stroke, a life-threatening condition characterized by core hyperthermia (≥40 °C), central nervous system dysfunction, and multi-organ failure with mortality rates of 20–70%, is increasingly recognized as a systemic inflammatory and hemodynamic disorder rather than a purely thermoregulatory collapse. Among the emerging mediators of its pathophysiology, trimethylamine N-oxide (TMAO)—a gut microbiota-derived metabolite generated from dietary choline, L-carnitine, and betaine—has attracted growing attention as a molecular link between heat-induced gut dysbiosis and multi-organ injury. This review synthesizes current evidence on the physiological and molecular mechanisms whereby TMAO is proposed to aggravate heat stroke pathology, including: (1) NLRP3 inflammasome activation via ROS-TXNIP, SIRT3-SOD2-mtROS, and cathepsin B-mediated pathways; (2) endothelial dysfunction through tight junction disruption, eNOS/NO suppression, and ALDH2/ROS/GSDMD-dependent pyroptosis; (3) platelet hyperreactivity and thrombosis through enhanced Ca^2+^ mobilization and IP_3_ signaling; (4) mitochondrial oxidative stress and bioenergetic failure; and (5) neuroinflammation via the gut–brain axis. We further discuss the concentration-dependent duality of TMAO as a chemical chaperone versus a pathological mediator, and evaluate translational strategies targeting TMAO metabolism, including gut microbiota modulation, TMA lyase inhibition, and NLRP3 blockade. Importantly, the majority of the mechanistic evidence synthesized herein derives from disease models other than heat stroke (e.g., atherosclerosis, chronic kidney disease, obesity, aging, preeclampsia, and ischemic stroke); direct evidence obtained in heat-stroke settings remains limited, and several proposed pathways should be regarded as hypotheses awaiting heat-stroke-specific validation. Elucidating TMAO-dependent mechanisms may therefore inform future biomarker development and therapeutic investigation—both of which require clinical validation in heat-stroke patients—in this increasingly prevalent climate-sensitive disease.

## 1 Introduction

Heat stroke is the most severe form of heat-related illness, resulting from exposure to high-temperature, high-humidity environments that overwhelm the body’s thermoregulatory capacity. It is defined by a core body temperature exceeding 40 °C accompanied by central nervous system dysfunction and multi-organ failure, representing a medical emergency with mortality rates of 20–70% (Bouchama et al., 2022; Eifling et al., 2024). Based on etiology, heat stroke is classified into exertional heat stroke (EHS), primarily affecting healthy young individuals engaged in strenuous physical activity in hot environments, and classic heat stroke (CHS), which predominantly affects the elderly, infants, and individuals with chronic diseases (Bouchama et al., 2022; Barletta et al., 2025). With global climate change and the increasing frequency of extreme heat events, the incidence and mortality of heat stroke are rising, posing a significant public health challenge (Romanello et al., 2023).

The pathophysiology of heat stroke is extraordinarily complex, involving direct thermal cytotoxicity, systemic inflammatory response syndrome (SIRS), disseminated intravascular coagulation (DIC), and multiple organ dysfunction syndrome (MODS) (Bouchama et al., 2022). Recent research has revealed a critical role for the gut microecology in heat stroke pathogenesis. Heat stress induces intestinal ischemia–reperfusion injury and barrier disruption, leading to gut microbiota dysbiosis and translocation of bacterial metabolites into the systemic circulation. These harmful metabolites further amplify systemic inflammation and organ injury (Nawaro et al., 2023; Mol et al., 2024; Wang et al., 2026).

Trimethylamine N-oxide (TMAO) is a gut microbiota-derived metabolite that has received considerable research attention in recent years. TMAO is produced through a two-step metabolic process: dietary precursors (choline, L-carnitine, and betaine) are first metabolized by gut bacterial TMA lyases to produce trimethylamine (TMA), which is then oxidized by hepatic flavin-containing monooxygenase 3 (FMO3) to TMAO (Bennett et al., 2013; Ufnal et al., 2015). Extensive evidence has established TMAO as a contributor to cardiovascular disease, chronic kidney disease, metabolic syndrome, and other conditions (Tang and Hazen, 2017; Janeiro et al., 2018). Importantly, emerging studies suggest that TMAO may play a role in the pathological progression of heat stroke (Mol et al., 2024; Wang et al., 2026). This review aims to systematically elucidate the molecular mechanisms through which TMAO contributes to heat stroke pathology, providing new theoretical foundations and potential therapeutic targets for heat stroke prevention and treatment.

## 2 Search strategy

We conducted a literature search of PubMed, Web of Science, and Scopus from database inception through June 2026 (last search date: 30 June 2026). The core search string combined TMAO-related and heat-illness-related terms: (“trimethylamine N-oxide” OR “TMAO” OR “trimethylamine oxide”) AND (“heat stroke” OR “heatstroke” OR “heat stress” OR “hyperthermia” OR “heat illness”); this was complemented by mechanism-focused searches pairing TMAO with “gut microbiota,” “NLRP3 inflammasome,” “endothelial dysfunction,” “platelet reactivity,” “thrombosis,” “mitochondrial dysfunction,” and “oxidative stress,” using Boolean operators (AND, OR) as appropriate for each database. No language restrictions were applied at the search stage; however, only English-language publications were ultimately retained, because the non-English records retrieved did not meet the relevance criteria below. Both original research articles and review papers were eligible; editorials, conference abstracts, and publications unrelated to TMAO biology or heat stress–related pathology were excluded. Titles and abstracts were screened for relevance to TMAO biology and its involvement in heat stress–related pathology, and the reference lists of included articles were manually checked for additional studies. This article is intended as a narrative review: the search was designed to identify representative and mechanistically informative publications rather than to exhaustively capture all eligible studies; accordingly, no systematic-review protocol (e.g., PRISMA), formal tally of identified and included studies, or risk-of-bias assessment was applied, and wording suggesting a systematic review has been avoided.

## 3 TMAO metabolism and biological properties

### 3.1 Biosynthetic pathway

TMAO is a low-molecular-weight organic compound with the chemical formula (CH_3_)_3_NO, belonging to the amine oxide class (Ufnal et al., 2015). Its biosynthesis involves a meta-organismal two-step pathway (**Figure 1**):

**Figure 1:**
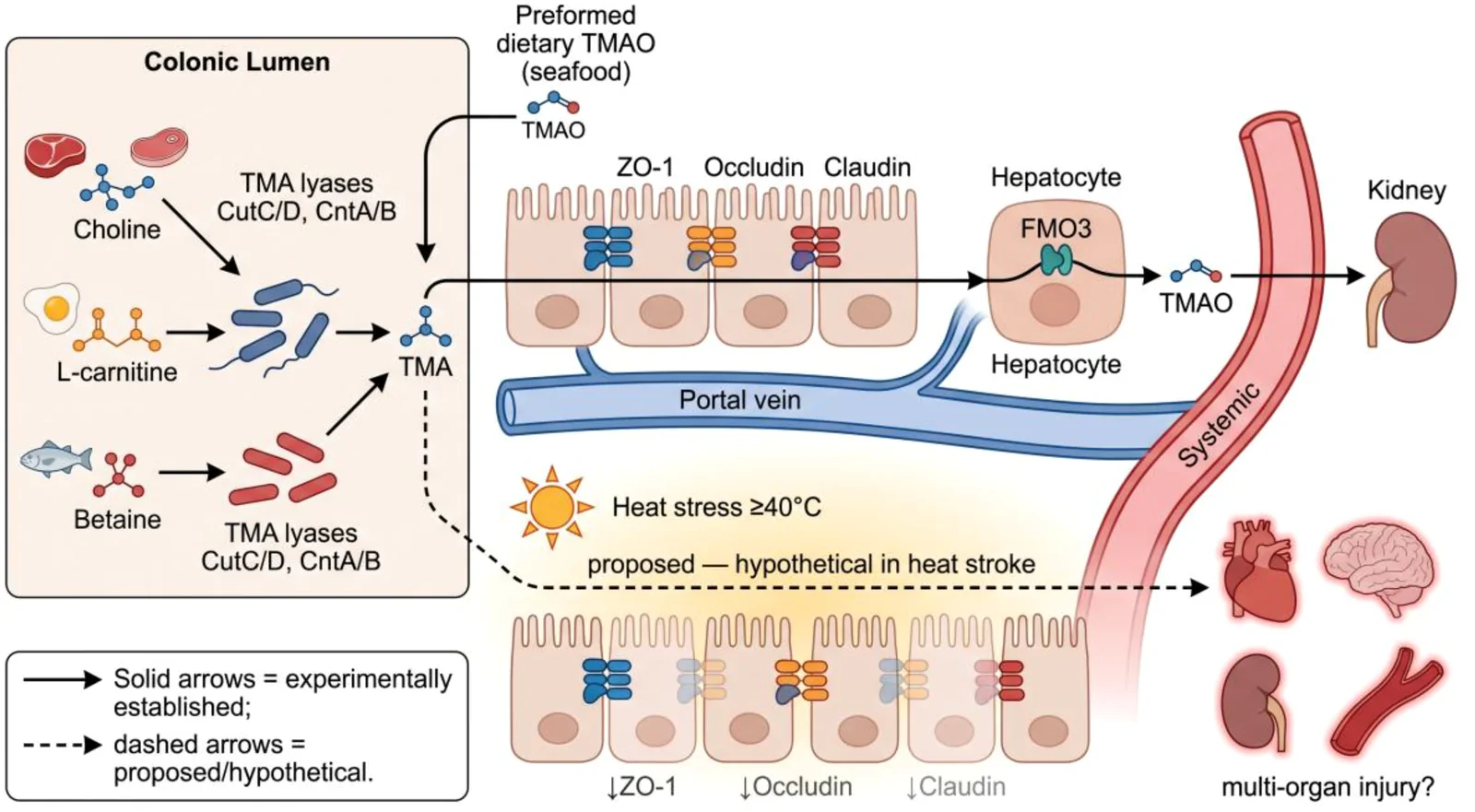
TMAO biosynthetic pathway and its proposed link to heat stroke. Dietary precursors (choline, L-carnitine, betaine) are metabolized by gut microbial TMA lyases (CutC/D, CntA/B) to trimethylamine (TMA), which is absorbed via the portal circulation and oxidized to TMAO by hepatic FMO3; preformed TMAO can also be absorbed directly from dietary sources (e.g., seafood), and circulating TMAO is cleared predominantly by the kidneys. Under heat stress, intestinal barrier disruption (↓ ZO-1, occludin, claudin) is proposed to enhance systemic entry of microbe-derived TMA (and possibly preformed TMAO); however, direct evidence that increased intestinal permeability raises circulating TMAO concentrations in heat stroke is currently lacking, and this step should be regarded as hypothetical. Solid arrows denote experimentally established relationships (in the indicated model systems); dashed arrows denote proposed or hypothetical links to heat-stroke pathology.

Step 1 (gut microbiota-dependent): Dietary precursors including choline, phosphatidylcholine, L-carnitine, betaine, and ergothioneine are metabolized in the colon by specific gut bacterial TMA lyases (notably the CutC/D enzyme system) to generate trimethylamine (TMA) (Wang et al., 2011; Ufnal et al., 2015). Foods rich in these precursors include red meat, egg yolks, seafood, and organ meats (Cho et al., 2017). Importantly, the human gut microbiota composition varies substantially between individuals, with omnivores harboring 2- to 5-fold higher abundance of TMA-producing Clostridiales species compared to vegetarians or vegans, translating to significantly elevated fasting and postprandial TMAO levels (De Filippis et al., 2016; Koeth et al., 2019).

Step 2 (liver-dependent): TMA is absorbed via the portal circulation and transported to the liver, where it is oxidized to odorless TMAO by the flavin-containing monooxygenase family, primarily FMO3 and secondarily FMO1 (Cashman and Zhang, 2002; Bennett et al., 2013). TMAO is subsequently released into the systemic circulation and is predominantly excreted via the kidneys (Hai et al., 2015). In patients with impaired renal function, TMAO clearance is markedly reduced, leading to plasma concentrations 20- to 40-fold higher than those observed in individuals with normal renal function, which may exacerbate systemic toxicity (Hai et al., 2015; Stubbs et al., 2016). In addition to this meta-organismal route, preformed TMAO present in certain foods—particularly saltwater fish—can be absorbed directly from the diet, bypassing microbial TMA production and hepatic oxidation (Cho et al., 2017).

### 3.2 Factors modulating TMAO levels

Circulating TMAO concentrations are regulated by multiple interacting factors that collectively determine an individual’s TMAO burden:

1. Dietary composition: Dietary intake of TMA precursors is the most direct modifiable determinant. Red meat, egg yolks, and saltwater fish are particularly rich sources of choline, phosphatidylcholine, and L-carnitine. A controlled dietary intervention demonstrated that chronic red meat consumption increases plasma TMAO levels approximately threefold compared to equivalent protein intake from white meat or non-meat sources, with levels normalizing within four weeks of dietary cessation (Wang et al., 2019). Mediterranean diet patterns, characterized by high polyphenol and fiber intake, are associated with lower TMAO levels, potentially through suppression of TMA-producing bacterial taxa (De Filippis et al., 2016).
2. Gut microbiota composition: The abundance and functional capacity of TMA-producing bacteria — predominantly within the Clostridiales order, including specific Firmicutes and Bacteroidetes species — vary substantially between individuals. L-carnitine supplementation in omnivorous, but not vegan, subjects produces measurable TMAO, demonstrating that existing microbial community structure determines metabolic output (Koeth et al., 2019). This interindividual variability suggests that gut microbiota profiling could identify individuals at elevated risk for TMAO-mediated heat stroke complications. Recent metagenomic studies further demonstrate that the presence of gut microbes harboring the *gbu* gene cluster — which encodes the enzymes catalyzing L-carnitine conversion to TMA — is a key determinant of TMAO production after L-carnitine intake, and that *gbu* gene abundance in the gut microbiota may serve as a biomarker for precision nutrition aimed at reducing TMAO burden (Wu et al., 2025).
3. Hepatic FMO3 activity: FMO3 expression is regulated by sex hormones (estrogen suppresses, testosterone upregulates), genetic polymorphisms, and bile acid signaling. Common FMO3 variants such as E158K and E308G significantly reduce enzyme activity, leading to diminished TMAO production but accumulation of foul-smelling TMA — the metabolic basis of trimethylaminuria (fish odor syndrome) (Cashman and Zhang, 2002; Bennett et al., 2013).
4. Renal function: The kidneys are the primary route of TMAO elimination. In chronic kidney disease, TMAO clearance decreases progressively with declining glomerular filtration rate, resulting in plasma concentrations 20- to 40-fold higher than normal (Hai et al., 2015; Stubbs et al., 2016). This is particularly relevant to heat stroke, as elderly individuals — the primary population affected by classic heat stroke — frequently have underlying renal impairment that may predispose them to pathological TMAO accumulation. Importantly, the relationship between TMAO and renal function is bidirectional: a large prospective community-based cohort demonstrated that elevated circulating TMAO independently predicts incident chronic kidney disease and accelerated kidney function decline, indicating that TMAO is not merely a marker of impaired clearance but also a contributor to renal injury progression (Wang et al., 2024a).
5. Age and sex: Plasma TMAO levels increase with age and are generally higher in males than premenopausal females, consistent with the regulatory effects of sex hormones on FMO3 expression (Ufnal et al., 2015; Canyelles et al., 2018; Janeiro et al., 2018).

### 3.3 Protein chaperone function

Notably, TMAO is evolutionarily an ancient osmolyte first discovered in marine elasmobranchs. TMAO functions as a “chemical chaperone,” stabilizing protein folding and counteracting denaturation induced by urea, high temperature, and high pressure (Yancey, 2005; Ufnal et al., 2015). In dogfish (Squalus acanthias) red blood cells, high concentrations of TMAO suppressed heat shock protein 70 (HSP70) induction while independently protecting cells from acute thermal stress (Kolhatkar et al., 2014). This property suggests that TMAO may exert a dual role — acting as a protein stabilizer at physiological concentrations while becoming pathological at supraphysiological levels.

## 4 The “gut-heat” axis: gut microbiota and TMAO in heat stroke

### 4.1 Heat stress-induced gut barrier disruption

The intestine is among the earliest organs affected in heat stroke. Rapid elevation of core body temperature triggers splanchnic vasoconstriction and intestinal ischemia–reperfusion injury, resulting in downregulation of tight junction proteins (zonula occludens-1 [ZO-1], occludin, claudin) and severe compromise of intestinal barrier integrity (Mol et al., 2024; Wang et al., 2026). This process constitutes what has been termed the “gastrointestinal paradigm” of heat stroke (Leon and Helwig, 2010). Importantly, TMAO itself directly contributes to intestinal barrier disruption: it dose-dependently downregulates occludin and claudin-1 expression in intestinal epithelial cells, increases paracellular permeability to 4-kDa FITC-dextran, and synergistically enhances bacterial protease-mediated cleavage of occludin (Chen et al., 2016; Nian et al., 2024). Elevated TMAO also activates the Toll-like receptor 4 (TLR4)/myeloid differentiation primary response 88 (MyD88)/nuclear factor kappa B (NF-κB) pathway in colonic epithelium, further promoting barrier dysfunction and creating a self-reinforcing cycle of permeability and inflammation (Nian et al., 2024). In colonic epithelial cells, TMAO has additionally been shown to prime NLRP3 inflammasome activation by suppressing ATG16L1-dependent autophagy, providing a further mechanism by which TMAO may sustain intestinal inflammation and barrier dysfunction (Yue et al., 2017).

### 4.2 Gut dysbiosis and enhanced TMAO production

Intestinal barrier disruption precipitates gut microbiota dysbiosis, characterized by significant compositional and functional alterations. Under heat stress conditions, the proportion of TMA-producing bacterial taxa (such as certain Bacteroidetes and Firmicutes) may increase, while beneficial commensal species — particularly Bifidobacterium and Lactobacillus — often decline, leading to elevated TMA/TMAO generation (Mol et al., 2024; Wang et al., 2026). The loss of microbial diversity further compounds this effect, as reduced competition for ecological niches allows TMA-producing opportunists to expand unchecked. Whether this compositional shift is accompanied by functional upregulation of microbial TMA lyase genes (cutC/D and cntA/B) in heat-stressed hosts has been proposed, but remains to be confirmed by direct metagenomic analyses in heat-stroke models (Wang et al., 2026).

Simultaneously, heat stress promotes translocation of lipopolysaccharide (LPS) and other bacterial products across the compromised intestinal barrier into the systemic circulation, establishing a “leaky gut” state (Nzvere et al., 2020; Wang et al., 2026). Whether TMAO itself translocates in excess across the heat-damaged barrier has not been directly demonstrated and therefore remains hypothetical. The translocation of LPS is particularly significant because it activates TLR4 signaling in multiple cell types, complementing TMAO’s pro-inflammatory effects and creating a synergistic inflammatory insult. The combination of increased TMAO production and enhanced intestinal permeability creates a feed-forward loop: TMAO itself further damages tight junctions and activates TLR4/NF-κB signaling in colonic epithelium (Nian et al., 2024), thereby worsening barrier dysfunction and potentially promoting further metabolite translocation. This self-amplifying cycle could hypothetically contribute to a sustained elevation of TMAO even after the initial thermal insult has resolved—a possibility that awaits confirmation by direct TMAO measurements, contributing to the prolonged multi-organ dysfunction observed in severe heat stroke cases.

### 4.3 Amplification cascade of harmful metabolites

Once in the circulation, TMAO synergizes with other gut-derived harmful substances, including LPS, to further aggravate systemic inflammation and organ injury. This vicious cycle — “gut barrier disruption → dysbiosis → metabolite translocation → organ damage → further barrier disruption” — is a critical driver of heat stroke progression to MODS (Wang et al., 2026). Evidence from cardiovascular and metabolic disease models demonstrates that TMAO promotes NLRP3 inflammasome–dependent inflammation, endothelial dysfunction, and platelet hyperreactivity (Section 5); by analogy, these actions are proposed to aggravate intravascular thrombosis and organ damage in heat stroke, although heat-stroke-specific interventional evidence is not yet available (Mol et al., 2024; Wang et al., 2026).

## 5 Molecular pathological mechanisms of TMAO in heat stroke

Before detailing these mechanisms, we emphasize that the evidence summarized below originates predominantly from cardiovascular, renal, metabolic, and neurological disease models rather than from experiments performed in heat-stroke settings. Throughout this section and in **Table 1**, direct heat-stroke evidence, indirect mechanistic evidence, and proposed pathways are therefore distinguished explicitly.

**Table 1:** Summary of TMAO-mediated pathological mechanisms proposed for heat stroke, with experimental models and evidence provenance.

Pathway	Key molecular targets	Cellular/tissue target	Mechanism	Evidence type	Refs	TMAO concentration & exposure	Direct heat-stroke evidence
NLRP3 inflammasome activation (ROS-TXNIP)	ROS, TXNIP, TRX, NLRP3, caspase-1, IL-1β, IL-18	Vascular endothelial cells	TMAO stimulates ROS → TXNIP dissociation from TRX → NLRP3 binding → inflammasome assembly	*In vitro* (HUVEC)	(Sun et al., 2016; Boini et al., 2017)	100–300 μM, 1–6 h (Sun et al., 2016); 30 μM, overnight (Boini et al., 2017)	No
NLRP3 inflammasome activation (SIRT3-SOD2)	SIRT3, SOD2, mtROS, NLRP3	Vascular endothelial cells	TMAO suppresses SIRT3 → SOD2 hyperacetylation → mtROS accumulation → NLRP3 activation	*In vitro* + animal model	(Chen et al., 2017)	150–900 μM; 24 h	No
NLRP3 inflammasome activation (cathepsin B)	Cathepsin B, NLRP3, lysosomes	Endothelial cells	TMAO increases cathepsin B activity → lysosomal damage → NLRP3 activation	*In vitro* (HUVEC) + mouse model	(Lei et al., 2023)	600 μM; 24 h	No
ER stress/trained immunity	PERK, IRE1α, ATF6, CREB	Aortic endothelial cells	TMAO upregulates ER stress sensors → mitochondrial ROS + glycolytic reprogramming → trained immunity	*In vitro* + transcriptomics	(Saaoud et al., 2023)	600 μM; 18–24 h	No
Endothelial tight junction disruption	ZO-1, occludin, claudin-1	Endothelial + intestinal epithelial cells	TMAO dose-dependently reduces tight junction proteins → increased permeability	*In vitro* + animal model	(Chen et al., 2016; Boini et al., 2017; Nian et al., 2024)	30 μM, overnight (Boini et al., 2017); *in vivo* (Chen et al., 2016; Nian et al., 2024)	No
eNOS/NO suppression	eNOS, NO, EDHF	Endothelial cells (rat aorta/femoral artery)	TMAO directly inhibits eNOS → reduced NO → impaired vasodilation; also suppresses EDHF-type relaxation	Ex vivo (vessel rings) + *in vitro*	(Sun et al., 2016; Matsumoto et al., 2020; Martelli et al., 2025)	300 μM, 6 h (Sun et al., 2016); 10–50 μM, 1 h (Martelli et al., 2025); 300 μM, 60 min (Matsumoto et al., 2020)	No
Pro-senescent effects (vascular aging)	GATA4	Endothelial cells	TMAO inhibits GATA4 degradation → vascular aging and endothelial senescence (CKD rats); not studied in heat stroke	Animal (CKD model)	(Zhong et al., 2025)	50–200 μM; 24–72 h	No
Pyroptosis	ALDH2, ROS, NLRP3, GSDMD	Vascular endothelial cells	TMAO inhibits ALDH2 → ROS accumulation → NLRP3 activation → GSDMD N-terminal pore formation → membrane perforation	*In vitro*	(Li et al., 2022)	300–900 μM; 24 h	No
Platelet hyperreactivity	Ca^2+^, IP_3_, PLC	Platelets	TMAO enhances Ca^2+^ mobilization via IP_3_ → augmented platelet activation to multiple agonists	Clinical (n > 4,000) + animal	(Zhu et al., 2016; Skye et al., 2018)	100 μM; 30 min (22 °C) (Zhu et al., 2016); *in vivo* (Skye et al., 2018)	No
Tissue factor upregulation	TF, NF-κB	Aortic endothelial cells	TMAO activates NF-κB → TF expression → enhanced extrinsic coagulation → arterial thrombosis	*In vitro* + animal model	(Witkowski et al., 2022)	200 μM; 2–6 h	No
Clopidogrel resistance	P2Y12, platelet aggregation	Platelets (rat)	Elevated TMAO attenuates clopidogrel antiplatelet efficacy → increased thrombotic recurrence	Animal (rat ischemia–reperfusion model)	(Ma et al., 2021)	500 mg/kg i.v.; *in vivo*	No
Mitochondrial dysfunction	SIRT3, SOD2, mtROS	Endothelial, cardiac, renal cells	TMAO suppresses SIRT3 → SOD2 hyperacetylation → mtROS accumulation (endothelium); ↓SOD2 → mitochondrial apoptosis (EPCs); renal tubular dysfunction/oxidative stress (obese mice); impaired pyruvate/fatty acid oxidation (cardiac)	*In vitro* + animal models	(Chen et al., 2017; Makrecka-Kuka et al., 2017; Sun et al., 2017; Shao et al., 2025)	150–900 μM, 24 h (Chen et al., 2017); 100–800 μM, 24 h (Shao et al., 2025); *in vivo* (Makrecka-Kuka et al., 2017; Sun et al., 2017)	No
mtDNA-cGAS-STING pathway	mtDNA, cGAS-STING	Osteoblastic cells (MC3T3-E1); renal/cardiac relevance extrapolated	mtDNA from injured mitochondria → cGAS–STING innate immune sensing (demonstrated in osteoblastic cells); plausible amplifier of systemic inflammation	Cell (osteoblastic MC3T3-E1)	(Sun et al., 2017; Li et al., 2026)	100–500 μM, 72 h (Li et al., 2026) (Sun et al., 2017); *in vivo*	No
EPC dysfunction	SOD2, apoptosis, autophagy	Endothelial progenitor cells	TMAO downregulates SOD2 → mitochondrial apoptosis + autophagic death → impaired vascular repair	*In vitro*	(Shao et al., 2025)	100–800 μM; 24 h	No
Neuroinflammation (gut-brain axis)	NLRP3, microglia, BBB	CNS (microglia, neurons)	TMAO crosses compromised BBB → activates microglial NLRP3 → neuroinflammation + cognitive deficits	Animal model	(Nam, 2019; Brunt et al., 2021; Wang et al., 2026)	N/A (*in vivo* (Brunt et al., 2021); reviews (Nam, 2019; Wang et al., 2026))	No
Vascular remodeling/atherosclerosis	NF-κB, inflammatory infiltration, smooth muscle apoptosis	Aortic wall, endothelium	TMAO promotes atherosclerosis and abdominal aortic aneurysm via inflammation + apoptosis	Animal + clinical	(Benson et al., 2023; Canyelles et al., 2023)	N/A (chronic model (Benson et al., 2023); review (Canyelles et al., 2023))	No
Pancreatic β-cell dysfunction	Mitochondria, ROS, glucose tolerance	Pancreatic β-cells	TMAO impairs β-cell function and glucose tolerance via mitochondrial damage and oxidative stress	Animal/*in vitro*	(Kong et al., 2024)	100 nM–10 μM; 18 h	No
Renal injury progression	GFR, kidney function	Kidney (tubulointerstitial, glomerular)	TMAO independently predicts incident CKD and accelerated kidney function decline	Clinical cohort	(Wang et al., 2024a; Su et al., 2025)	N/A (clinical cohort (Wang et al., 2024a); review (Su et al., 2025))	No

NR = not reported in an extractable form in the cited source. “Direct Heat-Stroke Evidence” indicates whether the finding was obtained in a heat-stroke or heat-stress experimental model; notably, none of the listed mechanisms has yet been demonstrated directly in heat-stroke models. HUVEC, human umbilical vein endothelial cells.

### 5.1 NLRP3 inflammasome activation and inflammatory cascades

The NLRP3 (NOD-like receptor family pyrin domain containing 3) inflammasome is a critical component of the innate immune system and plays a central role in the inflammatory storm of heat stroke. TMAO activates the NLRP3 inflammasome through multiple signaling pathways:

1. ROS-TXNIP-NLRP3 pathway: TMAO stimulates reactive oxygen species (ROS) production in vascular endothelial cells, causing thioredoxin-interacting protein (TXNIP) to dissociate from thioredoxin (TRX). Liberated TXNIP directly binds NLRP3, triggering inflammasome assembly, caspase-1 activation, and maturation and secretion of IL-1β and IL-18 (Sun et al., 2016; Boini et al., 2017). ROS scavenger N-acetylcysteine (NAC) or siRNA-mediated knockdown of TXNIP/NLRP3 significantly reverses TMAO-mediated inflammatory effects (Sun et al., 2016).
2. SIRT3-SOD2-mtROS pathway: TMAO suppresses expression and activity of the mitochondrial deacetylase sirtuin 3 (SIRT3), leading to increased manganese superoxide dismutase 2 (SOD2) acetylation and decreased enzyme activity, consequently causing mitochondrial ROS (mtROS) accumulation and NLRP3 inflammasome activation (Chen et al., 2017). This pathway plays a key role in TMAO-induced vascular inflammation.
3. Cathepsin B-mediated pathway: TMAO additionally increases the activity and expression of lysosomal cathepsin B, activating the NLRP3 inflammasome through lysosomal damage (Lei et al., 2023). The NLRP3-specific inhibitor MCC950 or cathepsin B inhibitors protect endothelial cells from TMAO-associated injury (Lei et al., 2023) (**Figure 2**).

**Figure 2:**
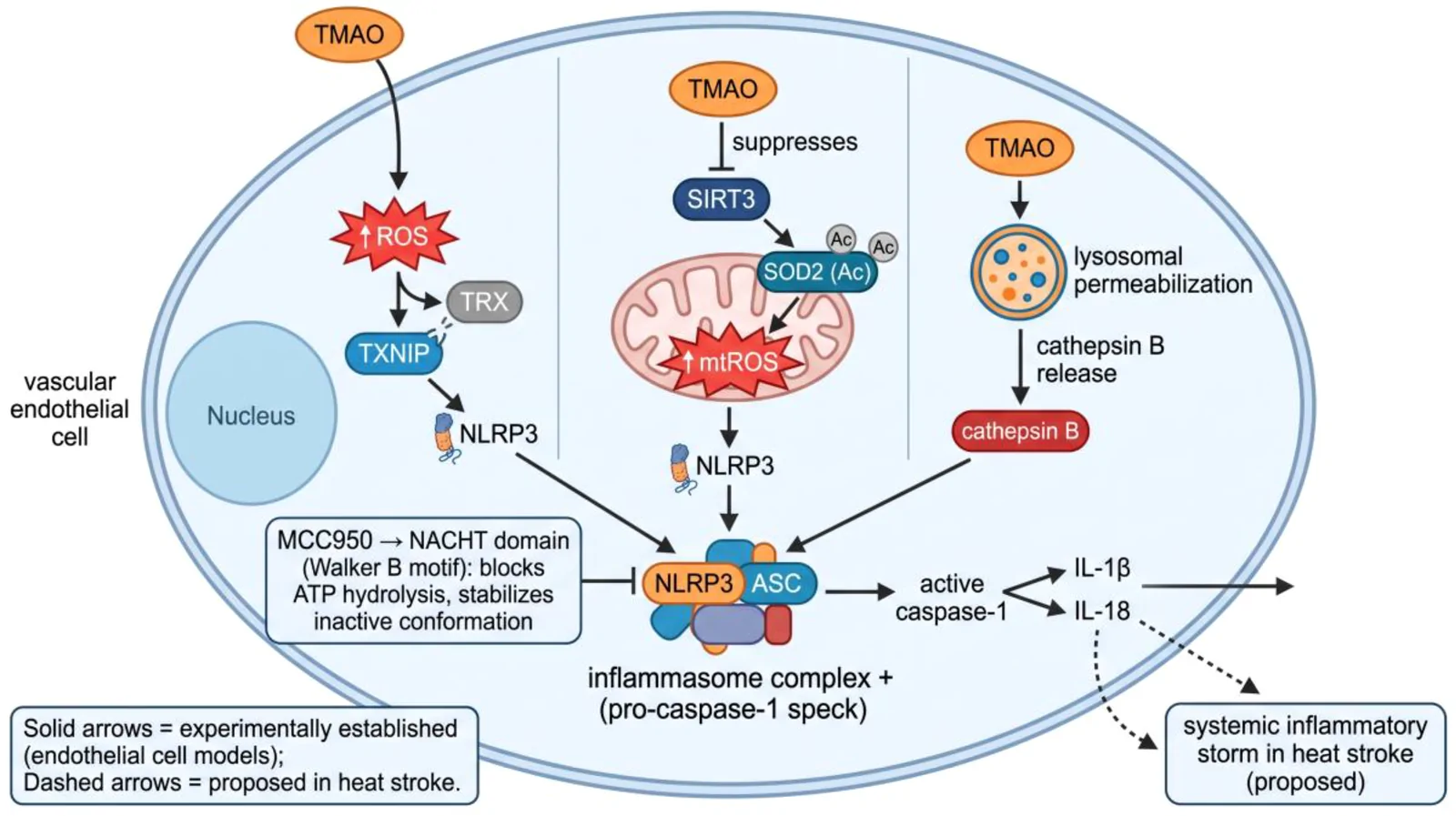
TMAO activates the NLRP3 inflammasome through multiple signaling pathways. Three distinct pathways: (1) ROS-TXNIP — TMAO-induced ROS releases TXNIP from TRX, enabling NLRP3 binding and caspase-1 activation; (2) SIRT3-SOD2-mtROS — TMAO suppresses SIRT3, causing SOD2 hyperacetylation, mtROS accumulation, and NLRP3 activation; (3) cathepsin B — TMAO induces lysosomal permeabilization and cathepsin B release. All converge on IL-1β/IL-18 maturation and systemic inflammation. Solid arrows denote experimentally established relationships (in the indicated model systems); dashed arrows denote proposed or hypothetical links to heat-stroke pathology.

Beyond these pathways, recent evidence has identified a fourth mechanism involving endoplasmic reticulum (ER) stress. TMAO upregulates ER stress sensors including protein kinase R-like ER kinase (PERK), inositol-requiring enzyme 1α (IRE1α), and activating transcription factor 6 (ATF6) in human aortic endothelial cells, with PERK acting as a putative TMAO receptor that integrates ER stress with mitochondrial ROS production through the PERK–cAMP response element-binding protein (CREB) signaling axis (Saaoud et al., 2023). Furthermore, TMAO induces trained immunity in endothelial cells — a phenomenon whereby innate immune cells develop memory-like characteristics — through combined ER stress, mitochondrial ROS, and metabolic reprogramming toward glycolysis (Saaoud et al., 2023). This trained immunity mechanism may be particularly relevant to heat stroke, wherein repeated sublethal heat exposures or prolonged hyperthermia could prime endothelial cells for exaggerated inflammatory responses upon subsequent TMAO exposure.

In the context of heat stroke, TMAO-driven NLRP3 inflammasome hyperactivation is proposed to contribute to the massive release of pro-inflammatory cytokines (IL-1β, IL-18, TNF-α, IL-6) and uncontrolled SIRS—a core driver of multi-organ damage—although direct confirmation in heat-stroke models is lacking (Leon and Helwig, 2010; Wang et al., 2026).

### 5.2 Endothelial dysfunction

The vascular endothelium, a continuous monolayer lining blood vessels, constitutes a critical barrier maintaining vascular permeability, regulating vascular tone, and preventing thrombosis. TMAO induces endothelial dysfunction through the following mechanisms:

1. Tight junction disruption: TMAO dose-dependently reduces ZO-1 expression in endothelial cells, increasing endothelial monolayer permeability and causing barrier dysfunction (Boini et al., 2017).
2. eNOS/NO pathway suppression: TMAO significantly inhibits endothelial nitric oxide synthase (eNOS) activity and nitric oxide (NO) production, impairing vasodilatory function (Sun et al., 2016; Querio et al., 2022).
3. Pro-senescent effects: In chronic disease settings, TMAO promotes vascular aging and endothelial senescence—for example, by inhibiting GATA4 degradation in the vasculature of chronic kidney disease rats (Zhong et al., 2025). Whether TMAO accelerates endothelial senescence in the context of heat stroke has not been studied; given the acute nature of heat stroke, senescence-related mechanisms are more likely to be relevant to delayed vascular complications than to the acute event (see Section 5.6).
4. Pyroptosis induction: Recent studies demonstrate that TMAO induces pyroptosis — a pro-inflammatory programmed cell death — in vascular endothelial cells through the aldehyde dehydrogenase 2 (ALDH2)/ROS/NLRP3/gasdermin D (GSDMD) signaling pathway, characterized by GSDMD N-terminal pore formation, plasma membrane perforation, and pro-inflammatory content release (Li et al., 2022) (**Figure 3**).

**Figure 3:**
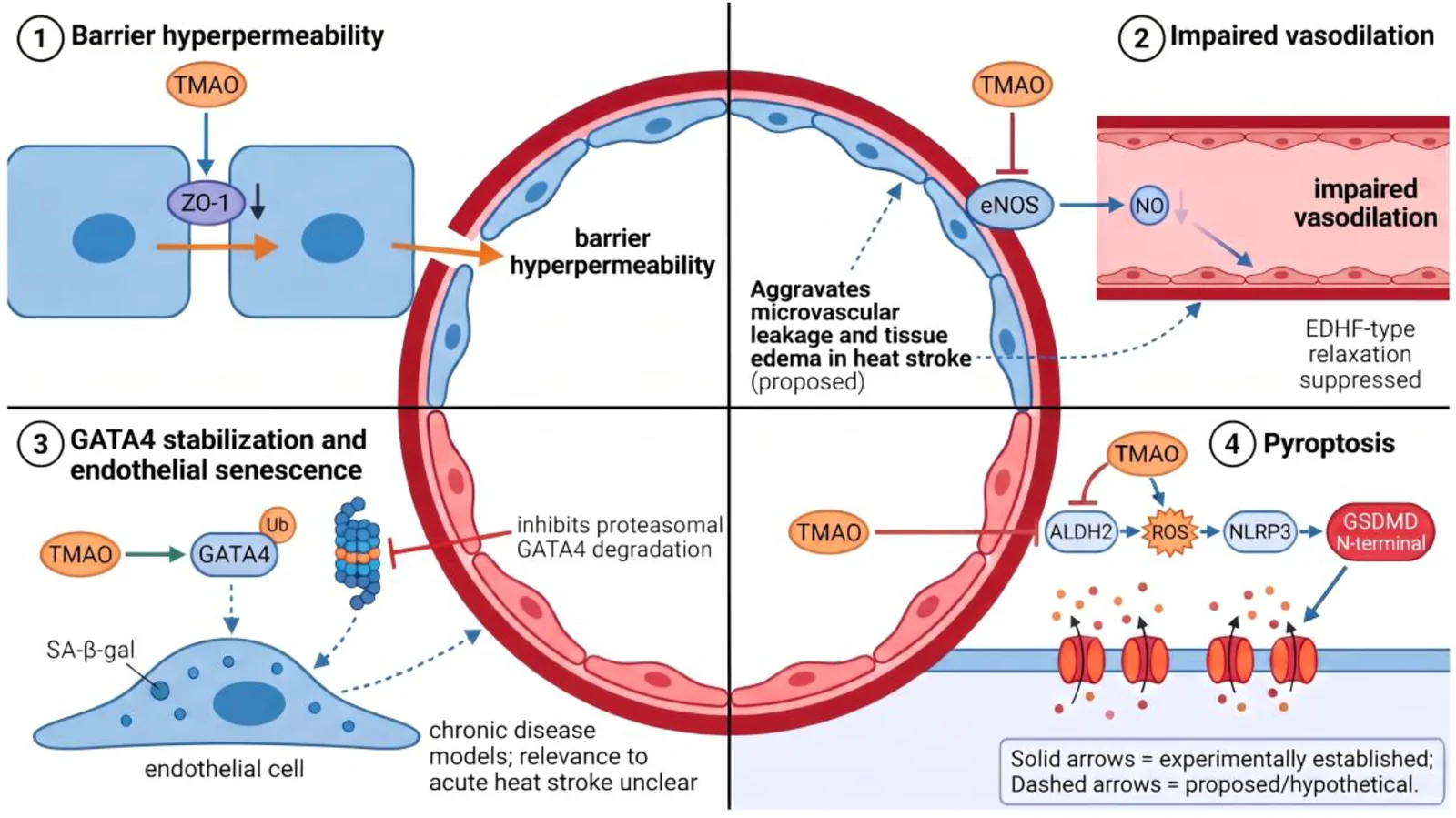
Molecular mechanisms of TMAO-induced endothelial dysfunction. (1) ZO-1 downregulation → barrier hyperpermeability; (2) eNOS/NO suppression → impaired vasodilation; (3) GATA4 stabilization → vascular aging and endothelial senescence (demonstrated in CKD rats; relevance to acute heat stroke unclear); (4) ALDH2/ROS/NLRP3/GSDMD-dependent pyroptosis. All mechanisms were characterized in non-heat-stroke endothelial models. Solid arrows denote experimentally established relationships (in the indicated model systems); dashed arrows denote proposed or hypothetical links to heat-stroke pathology.

Additionally, TMAO impairs endothelium-dependent vasodilation through non-NO mechanisms. In isolated rat femoral arteries, TMAO specifically suppresses endothelium-derived hyperpolarizing factor (EDHF)-type relaxation without affecting NO-mediated relaxation, suggesting that TMAO targets multiple vasodilatory pathways simultaneously (Matsumoto et al., 2020). Complementary findings demonstrate that TMAO acts as a direct inhibitor of eNOS, reducing NO production and acetylcholine-mediated vasorelaxation in rat aortas, at concentrations of 10–50 μM (1-h incubation) — a range achievable in pathological states (Martelli et al., 2025). The ω-3 polyunsaturated fatty acid docosahexaenoic acid (DHA) has been shown to alleviate TMAO-mediated impairment of neovascularization in endothelial progenitor cells by restoring mitochondrial function and reducing oxidative stress, highlighting a potential dietary protective strategy (Syu et al., 2023). Time-resolved transcriptomic and metabolomic analyses further reveal that TMAO induces distinct molecular signatures at 24 h versus 48 h of exposure: early responses involve TNF signaling suppression, while prolonged exposure activates cytokine-cytokine receptor interactions, arachidonic acid metabolism, and prostaglandin synthesis — suggesting a biphasic endothelial response to TMAO (Shanmugham et al., 2023).

Vascular remodeling and atherosclerosis: Beyond acute endothelial injury,TMAO has been implicated as a likely causal contributor to atherosclerotic cardiovascular disease, with clinical and genetic evidence supporting a dose-dependent relationship between circulating TMAO and coronary atherosclerotic burden (Canyelles et al., 2023). In a murine model, TMAO contributes to abdominal aortic aneurysm formation through NF-κB-mediated inflammatory cell infiltration and smooth muscle cell apoptosis, expanding the spectrum of TMAO-mediated vascular pathology beyond atherosclerosis to aneurysmal remodeling (Benson et al., 2023). Furthermore, TMAO has been identified as a therapeutic target in preeclampsia, a pregnancy-specific hypertensive disorder characterized by generalized endothelial dysfunction, with elevated TMAO levels correlating with disease severity and placental vascular injury (Wang et al., 2024b). These findings collectively indicate that TMAO-induced endothelial injury represents a common pathogenic mechanism across diverse vascular disorders, reinforcing its relevance to heat stroke-associated vascular damage.

In heat stroke, elevated circulating TMAO is proposed to synergize with direct thermal endothelial injury, potentially exacerbating barrier dysfunction, microvascular leakage, tissue edema, and multi-organ dysfunction; this proposed synergy awaits direct testing in heat-stroke models (Mol et al., 2024; Wang et al., 2026).

### 5.3 Platelet hyperreactivity and disseminated intravascular coagulation

DIC is among the most lethal complications of heat stroke and a key contributor to multi-organ failure and death. TMAO plays a significant role in coagulation derangement:

1. Enhanced platelet reactivity: Large-scale clinical studies (n > 4,000) have demonstrated that plasma TMAO levels independently predict incident (3-year) thrombotic event risk (myocardial infarction and stroke) (Zhu et al., 2016). Mechanistically, TMAO enhances stimulus-dependent Ca^2+^ release from platelet intracellular stores, increasing platelet responsiveness to multiple agonists (ADP, thrombin, collagen, arachidonic acid) even under submaximal stimulation (Zhu et al., 2016; Skye et al., 2018).
2. Inositol 1,4,5-trisphosphate (IP_3_) signaling pathway: TMAO enhances phospholipase C activity, increasing IP_3_ generation; IP_3_ binding to endoplasmic reticulum IP_3_ receptors triggers Ca^2+^ release, promoting platelet activation (Zhu et al., 2016; Skye et al., 2018). The effect of TMAO on platelet function is dose-dependent and rapidly reversible (Zhu et al., 2016).
3. Clopidogrel resistance: Experimental studies indicate that elevated TMAO levels attenuate the antiplatelet efficacy of clopidogrel, inducing clopidogrel resistance in a rat ischemia–reperfusion model (Ma et al., 2021).

A critical molecular link between TMAO and thrombosis involves vascular endothelial tissue factor (TF). Witkowski et al. demonstrated that TMAO upregulates TF expression and procoagulant activity in human aortic endothelial cells through activation of the NF-κB pathway, and that TMAO-induced TF contributes to enhanced arterial thrombus formation in a murine carotid artery injury model (Witkowski et al., 2022). This finding provides a direct mechanistic connection between TMAO-driven endothelial activation and the extrinsic coagulation pathway, which is particularly relevant to heat stroke where endothelial TF exposure is a primary driver of DIC initiation. Large prospective cohort studies have established that elevated plasma TMAO levels are dose-dependently associated with incident major adverse cardiovascular events, supporting the clinical significance of TMAO as a pro-thrombotic metabolite (Tang et al., 2013).

In heat stroke, heat injury–activated coagulation cascades are proposed to synergize with TMAO-induced platelet hyperreactivity, potentially accelerating progression from microvascular thrombosis to consumptive coagulopathy and DIC; however, this synergy has not been tested experimentally in heat-stroke models (Mol et al., 2024; Wang et al., 2026) (**Figure 4**).

**Figure 4:**
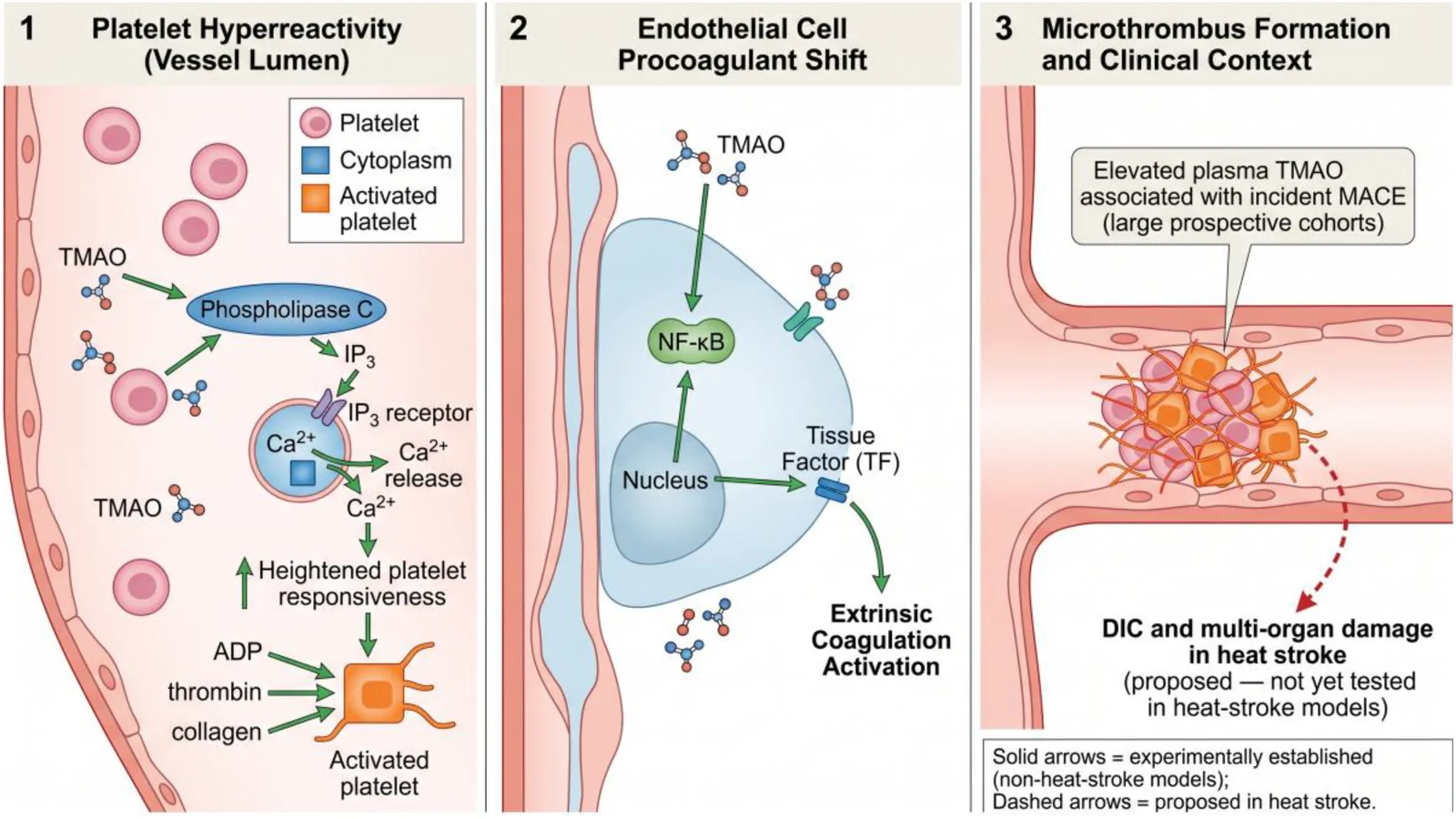
Proposed role of TMAO in heat stroke–associated vascular thrombosis and organ damage. TMAO enhances platelet Ca^2+^ release via IP_3_ signaling, increasing responsiveness to ADP, thrombin, and collagen (established in non-heat-stroke models). Synergy with heat-induced coagulation cascade activation is proposed to drive DIC—a leading cause of heat stroke mortality—but remains to be demonstrated in heat-stroke models. Solid arrows denote experimentally established relationships (in the indicated model systems); dashed arrows denote proposed or hypothetical links to heat-stroke pathology.

### 5.4 Mitochondrial dysfunction and oxidative stress

Mitochondria are primary targets of heat stress injury. TMAO is proposed to aggravate heat stroke–associated mitochondrial damage and oxidative stress through the following mechanisms, which have been characterized in non-heat-stroke models (**Figure 5**):

**Figure 5:**
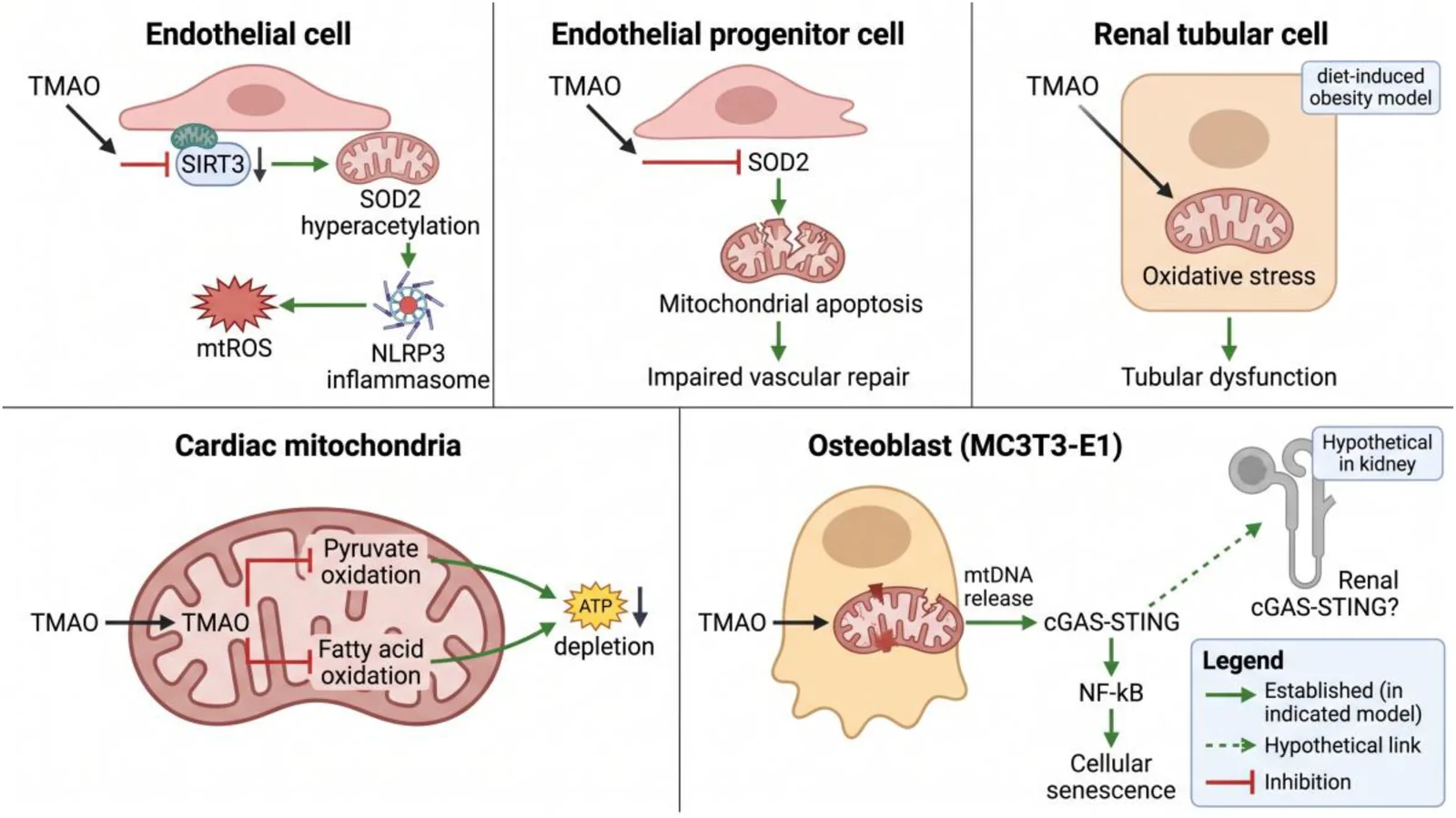
Proposed mechanisms of TMAO-mediated mitochondrial dysfunction and oxidative stress relevant to heat stroke (characterized in non-heat-stroke models): (1) SIRT3–SOD2–mtROS axis (endothelial cells); (2) SOD2 suppression → mitochondrial apoptosis (endothelial progenitor cells); (3) renal tubular dysfunction and oxidative stress (diet-induced obesity model); (4) impaired pyruvate/fatty acid oxidation → ATP depletion (cardiac mitochondria); (5) mtDNA → cGAS–STING innate immune sensing (osteoblastic MC3T3-E1 cells); renal cGAS–STING involvement is shown only as a hypothetical extrapolation from the osteoblastic evidence (dashed arrow). Solid arrows denote experimentally established relationships (in the indicated model systems); dashed arrows denote proposed or hypothetical links to heat-stroke pathology.

1. Mitochondrial oxidative stress and dysfunction: TMAO suppresses the mitochondrial deacetylase SIRT3, leading to SOD2 hyperacetylation, mitochondrial ROS (mtROS) accumulation, and NLRP3 inflammasome activation in endothelial cells (Chen et al., 2017); in endothelial progenitor cells, TMAO downregulates SOD2 (MnSOD), triggering mitochondrial apoptosis, pro-inflammatory activation, and impaired vascular repair capacity (Shao et al., 2025).
2. Renal mitochondrial injury: In a mouse model of diet-induced obesity, TMAO contributes to renal dysfunction and tubular oxidative stress (Sun et al., 2017); whether similar mitochondrial injury contributes to the acute kidney injury frequently observed in heat stroke remains to be determined.
3. Bioenergetic failure: TMAO impairs pyruvate and fatty acid oxidation in cardiac mitochondria, reducing ATP-generating capacity (Makrecka-Kuka et al., 2017). Consistent with heat-induced metabolic stress, exertional heat stroke in mice produces delayed myocardial metabolic disturbances—including glycolytic and tricarboxylic acid cycle disruption and accumulation of free fatty acids and bioactive lipids (Murray et al., 2022; Garcia et al., 2023); whether gut-derived TMAO contributes to these post-heat-stroke metabolic changes is a plausible but untested hypothesis.

Recent advances in the gut-heart axis further establish gut-derived metabolites, including TMAO, as contributors to heart failure pathogenesis and progression through systemic inflammation, endothelial dysfunction, and direct myocardial metabolic toxicity, providing a broader framework for understanding TMAO’s cardiac involvement in heat stroke (Snelson et al., 2025).

Beyond endothelial and myocardial mitochondria, TMAO-induced mitochondrial dysfunction may have systemic consequences: mitochondrial DNA (mtDNA) released from injured mitochondria acts as a damage-associated molecular pattern (DAMP) that can engage innate immune sensing via the cyclic GMP–AMP synthase–stimulator of interferon genes (cGAS–STING) pathway—a link between TMAO and cGAS–STING signaling recently demonstrated in osteoblastic cells (Li et al., 2026)—providing a plausible route by which TMAO-induced mitochondrial injury could amplify systemic inflammation in severe heat stroke; this mechanism has not been tested in heat-stroke models.

Renal involvement may further amplify this systemic process. TMAO has been implicated in the development and progression of kidney disease, including renal oxidative stress and tubular injury (Su et al., 2025). Because acute kidney injury is a frequent complication of severe heat stroke, reduced renal TMAO clearance together with renal mitochondrial injury could establish a self-reinforcing cycle that sustains elevated circulating TMAO and aggravates multi-organ damage; this interaction has not been directly examined in heat-stroke models.

Pancreatic β-cell dysfunction: A recent high-impact study demonstrates that TMAO impairs pancreatic β-cell function and glucose tolerance through mitochondrial dysfunction and oxidative stress, providing direct evidence that TMAO-mediated mitochondrial damage extends beyond the vasculature to metabolic organs (Kong et al., 2024). This finding is clinically relevant to heat stroke, in which hyperglycemia and impaired insulin sensitivity are common complications that aggravate systemic inflammation and worsen clinical outcomes.

### 5.5 Central nervous system injury

Heat stroke is frequently accompanied by severe central nervous system dysfunction, manifesting as delirium, seizures, and coma, with some survivors experiencing long-term cognitive deficits (He et al., 2023). TMAO may affect the CNS through the gut-brain axis (Wang et al., 2026) (**Figure 6**).

**Figure 6:**
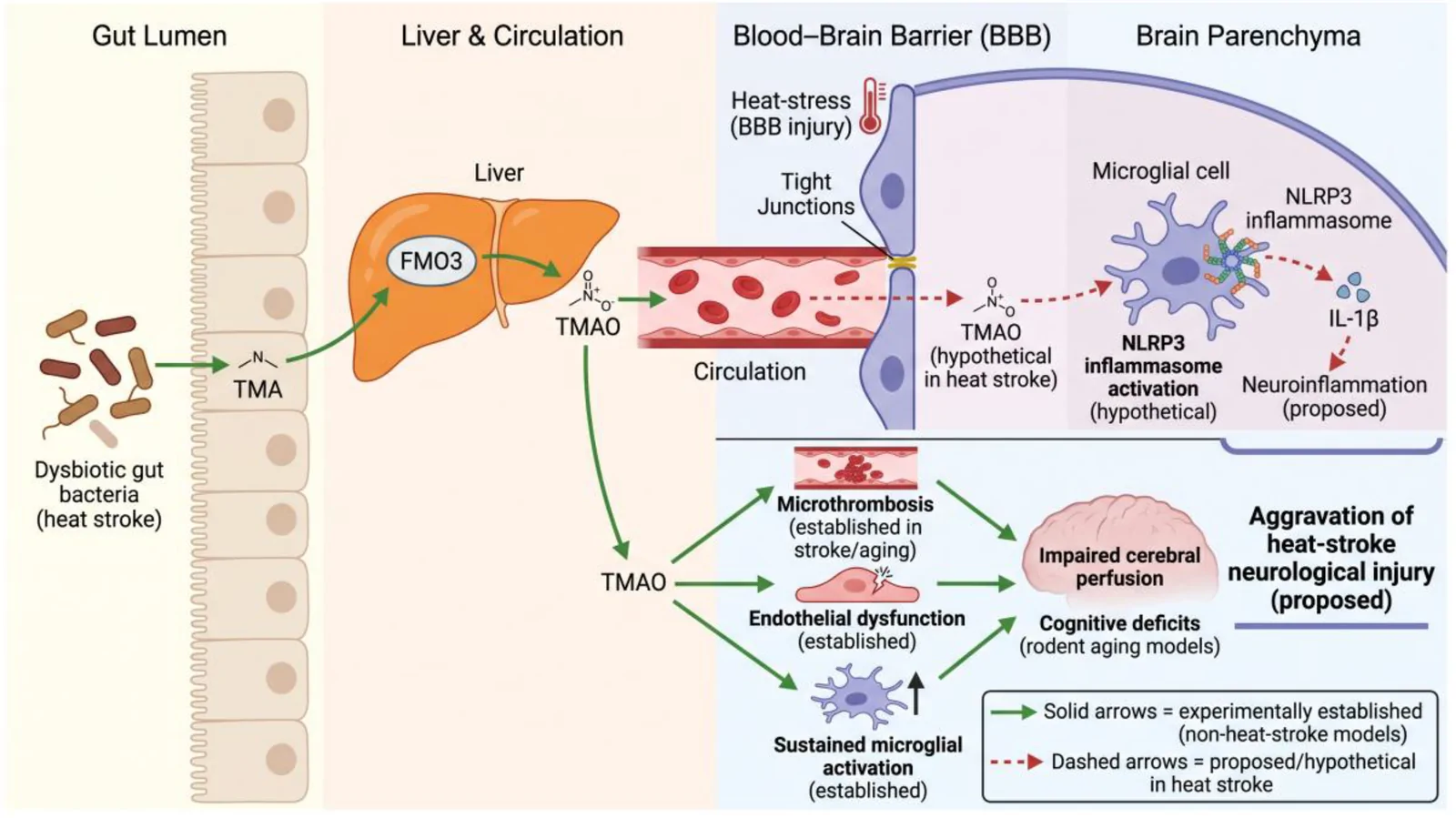
Proposed impact of TMAO on the CNS through the gut–brain axis. Gut-derived TMAO may cross the heat-compromised blood–brain barrier and activate microglial NLRP3 inflammasomes (hypothetical in heat stroke), while systemic endothelial dysfunction and microthrombosis impair cerebral perfusion. Solid arrows denote experimentally established relationships (in the indicated model systems); dashed arrows denote proposed or hypothetical links to heat-stroke pathology.

Gut-derived TMAO produced after dysbiosis and barrier disruption enters the systemic circulation and subsequently crosses the compromised blood-brain barrier into brain tissue. It has been proposed that TMAO may then activate microglial NLRP3 inflammasomes, thereby promoting neuroinflammation and aggravating heat stroke–associated neural injury, although direct evidence in heat-stroke models is currently lacking (Wang et al., 2026). Additionally, TMAO-induced endothelial dysfunction and microthrombosis could further exacerbate cerebral microcirculatory impairment and brain tissue ischemia–hypoxia. Emerging evidence indicates that TMAO contributes specifically to stroke pathology: elevated circulating TMAO levels are associated with increased ischemic stroke risk and worse neurological outcomes, potentially through enhanced platelet-neutrophil aggregate formation in cerebral microvessels and promotion of blood-brain barrier disruption (Nam, 2019). In the context of heat stroke-related neurological injury, TMAO may also exacerbate excitotoxicity by impairing astrocyte glutamate reuptake, although direct evidence for this mechanism remains limited and requires further investigation (Janeiro et al., 2023). Furthermore, TMAO-mediated neuroinflammation may persist beyond the acute heat stroke episode: rodent models demonstrate sustained microglial activation and hippocampal-dependent cognitive deficits weeks after TMAO exposure, raising concerns about long-term neurological sequelae in heat stroke survivors with chronically elevated TMAO (Brunt et al., 2021). Recent reviews further consolidate the evidence linking TMAO to ischemic stroke: mechanistic and clinical data indicate that TMAO increases stroke risk through promotion of platelet activation, endothelial dysfunction, and blood-brain barrier disruption, with circulating TMAO levels correlating with infarct volume and functional outcomes (Dolkar et al., 2024; Li et al., 2024a). These findings strengthen the rationale for investigating TMAO as a modifiable mediator of heat stroke-associated cerebral injury. **Table 1** summarizes the key molecular pathological mechanisms of TMAO across all affected organs in heat stroke.

### 5.6 TMAO in heat stroke: cause, consequence, or marker?

A central unresolved question is whether elevated TMAO is a driver of heat-stroke pathology or, at least in part, a consequence of it. Acute kidney injury is common in severe heat stroke, and impaired renal clearance can increase circulating TMAO independently of increased microbial production (Hai et al., 2015; Stubbs et al., 2016). Diet, age, baseline renal function, medication use, hepatic FMO3 activity, and microbiota composition are additional important determinants of TMAO levels (Cashman and Zhang, 2002; Bennett et al., 2013; Ufnal et al., 2015). TMAO may therefore represent, in some contexts, a marker of renal or systemic metabolic dysfunction rather than an independent driver of organ injury; prospective studies measuring serial TMAO concentrations together with renal function in heat-stroke patients are needed to disentangle these possibilities.

The temporal dimension of the proposed mechanisms also warrants emphasis. Heat stroke is an acute condition that evolves over hours, whereas several TMAO-associated processes reviewed above—including endothelial senescence, vascular remodeling, trained immunity, and atherosclerosis—have primarily been studied after weeks to years of exposure (Benson et al., 2023; Canyelles et al., 2023; Saaoud et al., 2023). It is unclear whether TMAO concentrations rise rapidly enough to engage these pathways during the acute phase of heat stroke. Mechanisms plausibly relevant to the acute event (e.g., NLRP3 inflammasome activation, endothelial barrier dysfunction, platelet hyperreactivity, and mitochondrial oxidative stress) should be distinguished from those more likely to contribute to recovery, delayed organ injury, or long-term complications (Nzvere et al., 2020).

Finally, the two clinical forms of heat stroke may differ substantially in TMAO kinetics and clinical meaning. Exertional heat stroke usually affects younger individuals and is frequently associated with intense physical activity, rhabdomyolysis, metabolic acidosis, and acute kidney injury, whereas classic heat stroke more often occurs in older patients with chronic diseases, impaired baseline renal function, altered dietary habits, and multiple medication exposures (Bouchama et al., 2022; Barletta et al., 2025). Because renal function strongly influences TMAO clearance (Hai et al., 2015; Stubbs et al., 2016), circulating TMAO profiles—and their interpretation as candidate biomarkers or therapeutic targets—are likely to differ between exertional and classic heat stroke and should be analyzed separately in future studies.

## 6 Dual role of TMAO: chemical chaperone vs. pathological mediator

A noteworthy aspect of TMAO in heat stroke is its concentration-dependent duality. At physiological concentrations, TMAO functions as a chemical chaperone with protein-stabilizing properties. In dogfish erythrocytes, TMAO suppresses HSP70 induction under heat stress while independently protecting cells from thermal injury, as evidenced by attenuated caspase 3/7 activation, reduced protein carbonylation, and preserved membrane integrity and hemoglobin oxygen affinity (Kolhatkar et al., 2014). This suggests that TMAO’s chemical chaperone function can substitute for molecular chaperone HSP70’s thermal protection. Recent molecular dynamics simulations provide atomic-level insight into this protective function, demonstrating that TMAO stabilizes native protein conformations by modulating backbone conformational entropy and reinforcing intra-molecular interactions, thereby counteracting heat-induced unfolding and aggregation (Boob et al., 2023). In deep-sea organisms, TMAO concentrations increase linearly with depth, counteracting protein denaturation under high hydrostatic pressure — an evolutionary adaptation that underscores its fundamental role as a cytoprotective osmolyte (Yancey, 2005).

However, whether circulating TMAO actually reaches pathogenic concentrations in heat stroke is unknown. The chemical-chaperone effect of TMAO has largely been demonstrated in marine organisms and experimental protein systems (Yancey, 2005; Kolhatkar et al., 2014; Boob et al., 2023), and the concentrations involved in those settings are not necessarily comparable to normal human plasma concentrations (typically ~1–5 μM in healthy adults, rising to 30–50 μM in renal impairment or cardiovascular disease (Hai et al., 2015; Stubbs et al., 2016)). Direct measurements of TMAO kinetics in heat-stroke patients or animal models are currently lacking.

Moreover, the concentrations reported for NLRP3 activation in endothelial cells (30–300 μM (Sun et al., 2016; Boini et al., 2017)) and for platelet hyperreactivity (~100 μM (Zhu et al., 2016)) were obtained in different experimental systems, exceed typical physiological plasma levels, and cannot be combined to define a single transition point between protective and harmful effects. The dual-role concept is therefore retained here as an unresolved, context-dependent hypothesis. Elucidating concentration–response relationships in heat-stroke-relevant settings should be a priority for future research, as this information is essential for defining therapeutic targets and biomarker cutoffs.

## 7 TMAO as a therapeutic target in heat stroke

Given TMAO’s proposed role in heat stroke pathogenesis, targeting TMAO metabolic pathways may represent a novel strategy for heat stroke prevention and treatment (**Figure 7**). It should be emphasized that none of the strategies discussed below—including DMB, IMC, FMC, FMO3 inhibition, MCC950, engineered probiotics, nanoparticle-based delivery, and fecal microbiota transplantation—has been validated for the treatment of heat stroke. These approaches are presented as future research directions rather than clinical recommendations. Their expected onset of action must also be weighed against the rapid progression of heat stroke, in which immediate cooling and organ support remain the only established life-saving interventions: slow-onset strategies (e.g., dietary or microbiota modulation) are conceptually better suited to prevention in at-risk populations, whereas fast-acting downstream blockade (e.g., NLRP3 inhibition) would be the more plausible adjunct—if ever validated—in the acute setting.

**Figure 7:**
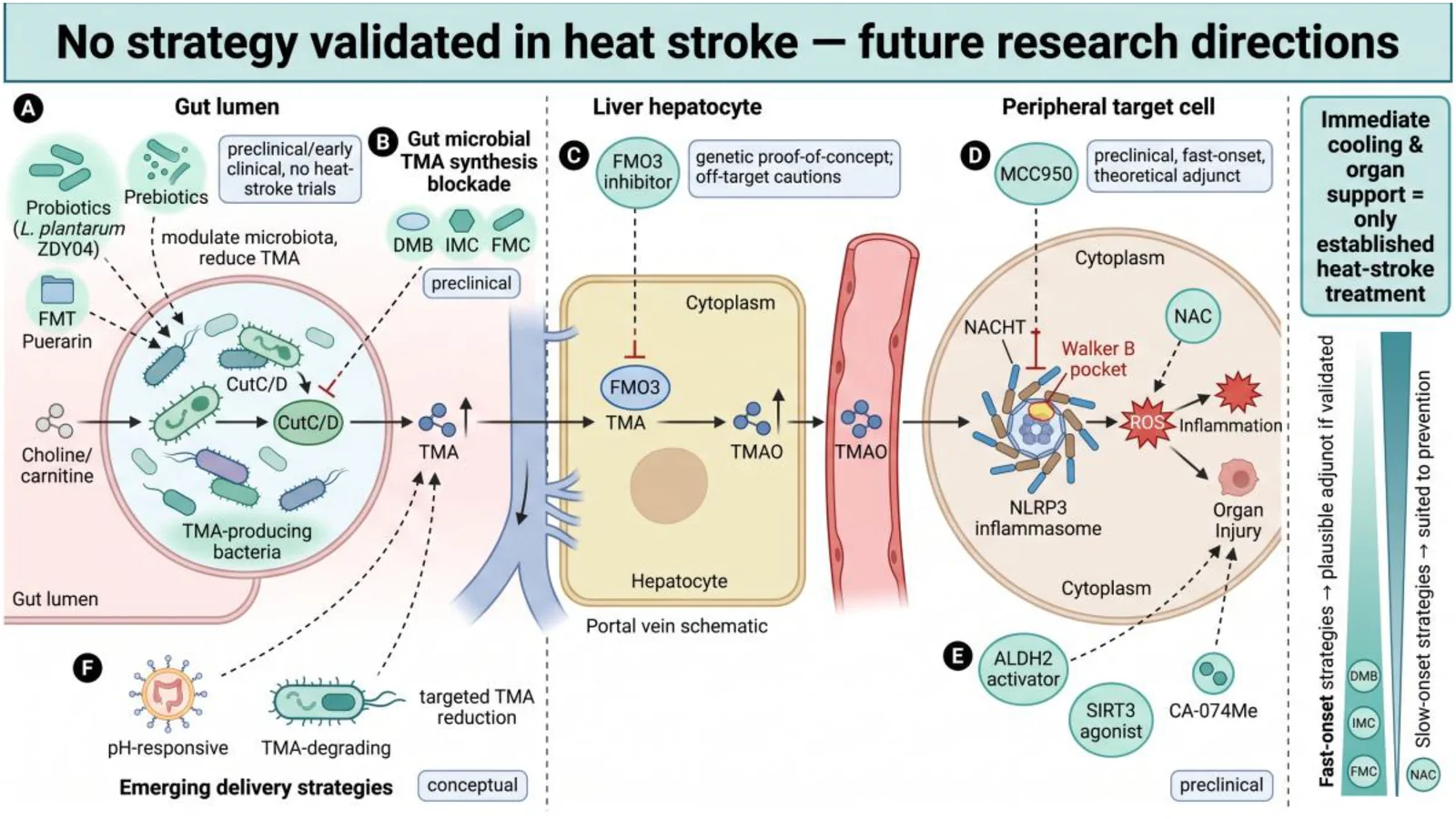
TMAO as a therapeutic target: integrated intervention strategies. Intervention points: **(A)** gut microbiota modulation; **(B)** TMA lyase inhibitors (DMB, IMC, FMC); **(C)** FMO3 inhibition; **(D)** NLRP3 blockade (MCC950, NAC); **(E)** downstream pathway inhibition (ALDH2 activators); **(F)** targeted intestinal delivery systems. None of these strategies has been validated in heat stroke; all represent future research directions. Solid arrows denote experimentally established relationships (in the indicated model systems); dashed arrows denote proposed or hypothetical links to heat-stroke pathology.

### 7.1 Gut microbiota modulation

Modulating gut microbiota composition to reduce TMA-producing bacterial abundance is a fundamental strategy for lowering TMAO levels. Probiotics, prebiotics, and synbiotic preparations may reduce TMAO production — and hypothetically its translocation across a compromised barrier — by restoring gut microbial homeostasis and enhancing intestinal barrier function (Mol et al., 2024; Wang et al., 2026). Specific probiotic strains have demonstrated TMAO-lowering efficacy: Lactobacillus plantarum ZDY04 lowers circulating TMAO levels in high-choline-fed mice by modulating gut microbiota composition (Qiu et al., 2018). Heat acclimatization preconditioning may exert protective effects through preserving intestinal barrier integrity and modulating gut microbiota structure (Wang et al., 2026), potentially representing a non-pharmacological approach to reducing TMAO-associated heat stroke risk in at-risk populations. Natural products targeting TMA-producing bacteria represent an emerging microbiota-based strategy: the isoflavone puerarin alleviates atherosclerosis by reducing the abundance of Prevotella copri and its TMA production, and a one-week oral intervention effectively lowered both P. copri levels and plasma TMAO concentrations in patients with carotid artery plaques (Li et al., 2024b). Antibiotic-mediated depletion of gut microbiota has also been shown to attenuate the transition from acute kidney injury to chronic kidney disease through inhibition of NADPH oxidase 2 and reduction of TMAO levels, although the risks of antibiotic use — including dysbiosis and antimicrobial resistance — necessitate cautious interpretation of this approach (Lee et al., 2024).

### 7.2 TMA lyase inhibition

3,3-Dimethyl-1-butanol (DMB): As a structural analog of choline, DMB competitively inhibits gut microbial TMA lyase activity, reducing TMA generation and consequently circulating TMAO levels (Wang et al., 2015). Animal studies confirm that DMB suppresses choline-enhanced platelet hyperreactivity and thrombosis potential (Zhu et al., 2016; Skye et al., 2018).

Iodomethylcholine (IMC) and fluoromethylcholine (FMC): These choline analogs similarly reduce TMAO through TMA lyase inhibition, decreasing platelet hyperreactivity and thrombotic risk (Roberts et al., 2018).

### 7.3 FMO3 targeting

FMO3 is the key hepatic enzyme catalyzing TMA oxidation to TMAO. Inhibiting FMO3 activity effectively reduces circulating TMAO levels while simultaneously increasing TMA, which can be excreted via breath and sweat (Cashman and Zhang, 2002; Bennett et al., 2013). Genetic studies provide proof-of-concept: individuals with loss-of-function FMO3 polymorphisms exhibit dramatically reduced plasma TMAO, confirming FMO3 as a rate-limiting enzyme in the TMAO biosynthetic pathway (Cashman and Zhang, 2002). However, FMO3 inhibition requires caution for several reasons. First, FMO3 participates in the metabolism of numerous xenobiotics and pharmaceuticals, including tamoxifen, benzydamine, and itopride, and its inhibition could alter drug pharmacokinetics and increase toxicity risk (Cashman and Zhang, 2002). Second, FMO3 metabolizes TMA to prevent its accumulation — unopposed TMA buildup causes trimethylaminuria, characterized by unpleasant body odor and potential neurologic effects at high concentrations. Third, FMO3 expression is sexually dimorphic, regulated by estrogen and testosterone, meaning that therapeutic targeting would need to account for sex-specific dosing considerations. Selective FMO3 inhibitors with tissue-specific delivery or temporary action during acute heat stroke episodes may represent a viable strategy, but no such compounds have been tested in heat stroke models to date. Notably, a recent study demonstrates that the FMO3/TMAO/HSP90β axis aggravates metabolic dysfunction-associated fatty liver disease by disrupting mitochondrial protein homeostasis, further underscoring the systemic metabolic consequences of FMO3-TMAO pathway activation and the broad therapeutic potential of its modulation (Guo et al., 2025).

### 7.4 Downstream inflammatory pathway inhibition

Given that TMAO exerts its major pro-inflammatory effects via NLRP3 inflammasome activation, NLRP3-specific inhibitors such as MCC950 hold potential for heat stroke treatment (Lei et al., 2023). In the context of heat stroke, MCC950 could interrupt the TMAO-driven inflammatory cascade at a critical convergence point, potentially limiting multi-organ damage regardless of upstream TMAO concentration (Lei et al., 2023). ROS scavengers such as NAC may also attenuate TMAO-mediated inflammation and endothelial injury through inhibition of the ROS-TXNIP-NLRP3 pathway (Sun et al., 2016; Boini et al., 2017). Additionally, SIRT3 agonists — including resveratrol and nicotinamide riboside — may counteract the SIRT3-SOD2-mtROS pathway by restoring mitochondrial deacetylase activity, thereby reducing mtROS accumulation and subsequent NLRP3 activation (Chen et al., 2017). The cathepsin B inhibitor CA-074Me represents another downstream target, blocking the lysosomal damage-mediated NLRP3 activation pathway (Lei et al., 2023). A multi-target approach combining upstream TMAO reduction with downstream inflammasome blockade may offer synergistic therapeutic benefit, particularly in severe heat stroke, where rapid intervention is critical should circulating TMAO prove to be markedly elevated. Mechanistically, MCC950 interacts with the Walker B region within the NACHT domain of NLRP3, inhibiting its ATPase activity and stabilizing the protein in an inactive conformation, thereby preventing NLRP3 activation and downstream caspase-1 activation (Coll et al., 2019; Tapia-Abellán et al., 2019); it has demonstrated efficacy in preclinical models of multiple inflammatory diseases.

### 7.5 Targeted delivery systems and emerging technologies

Emerging microencapsulation, nanotechnology, and mucosal adhesion technologies offer novel approaches for gut-targeted therapeutic delivery, potentially enabling precise modulation of gut microbiota and TMAO production (Wang et al., 2026). pH-responsive nanoparticles that release TMA lyase inhibitors specifically in the colon could minimize systemic off-target effects while maximizing local microbial modulation. Additionally, engineered probiotic bacteria expressing TMA-degrading enzymes represent a next-generation strategy: synthetic biology approaches could create live biotherapeutic products that constitutively convert TMA to inert metabolites within the gut lumen, providing sustained TMAO reduction without requiring daily drug administration. Fecal microbiota transplantation (FMT), while still experimental for heat stroke indications, offers a broader approach to correcting the dysbiotic gut microbiota profile that underlies TMAO overproduction. In clinical settings, biomarker-guided therapy — using rapid point-of-care TMAO quantification to identify patients who would benefit most from TMAO-targeted interventions — could enable precision medicine approaches to heat stroke treatment. However, all of these strategies remain at preclinical or early translational stages, and their efficacy and safety in heat stroke models require systematic evaluation before clinical application. **Table 2** summarizes the therapeutic strategies targeting TMAO discussed above, along with their evidence levels and limitations.

**Table 2:** Therapeutic strategies targeting TMAO in heat stroke.

Strategy	Agent/approach	Mechanism of action	Evidence level	Limitations	Refs
Gut microbiota modulation	*L. plantarum* ZDY04; probiotics; prebiotics; FMT	Competitive exclusion of TMA-producing bacteria; restoration of tight junctions; enhanced barrier function	Preclinical (animal)	Strain specificity; dose optimization needed; no heat stroke trials	(Qiu et al., 2018; Mol et al., 2024; Wang et al., 2026)
Dietary intervention	Mediterranean diet; reduced red meat/egg intake; DHA supplementation	Reduced TMA precursor intake; suppression of TMA-producing taxa; DHA restores EPC mitochondrial function	Clinical (observational) + preclinical	Dietary compliance; slow onset; DHA efficacy only in EPC models	(De Filippis et al., 2016; Wang et al., 2019; Syu et al., 2023)
TMA lyase inhibition	DMB (3,3-dimethyl-1-butanol)	Structural choline analog; competitively inhibits CutC/D TMA lyase → reduced TMA generation	Preclinical (animal)	Non-selective; potential off-target effects; pharmacokinetics unclear	(Wang et al., 2015; Zhu et al., 2016; Skye et al., 2018)
TMA lyase inhibition	IMC (iodomethylcholine); FMC (fluoromethylcholine)	Choline analogs; potent TMA lyase inhibition → reduced platelet hyperreactivity	Preclinical (animal)	Limited safety data; iodine load concern (IMC)	(Roberts et al., 2018)
FMO3 targeting	FMO3 inhibitors (experimental)	Blocks hepatic TMA → TMAO oxidation	Genetic (human proof-of-concept)	Off-target drug metabolism; TMA accumulation; sex-specific regulation	(Cashman and Zhang, 2002; Bennett et al., 2013)
NLRP3 inhibition	MCC950	Binds the Walker B motif within the NACHT domain → blocks ATP hydrolysis → stabilizes inactive NLRP3 conformation	Preclinical (multiple models)	No heat stroke trials; potential immunosuppression	(Coll et al., 2019; Tapia-Abellán et al., 2019; Lei et al., 2023)
ROS scavenging	N-acetylcysteine (NAC)	Scavenges ROS → prevents TXNIP-NLRP3 activation	*In vitro* + clinical (other indications)	Non-specific; high doses needed; limited bioavailability	(Sun et al., 2016; Boini et al., 2017)
SIRT3 activation	Resveratrol; nicotinamide riboside	Restores SIRT3 deacetylase activity → reduces SOD2 acetylation → attenuates mtROS	Preclinical	Bioavailability issues; pleiotropic effects	(Chen et al., 2017)
Cathepsin B inhibition	CA-074Me	Blocks lysosomal cathepsin B → prevents NLRP3 activation via lysosomal damage	*In vitro*	Limited *in vivo* data; specificity concerns	(Lei et al., 2023)
Nanotechnology	pH-responsive nanoparticles; engineered probiotics	Colon-targeted TMA lyase inhibitor delivery; synthetic TMA-degrading bacteria	Conceptual/early preclinical	No heat stroke data; regulatory challenges	(Wang et al., 2026)
Heat acclimatization	Gradual thermal preconditioning	Preserves intestinal barrier integrity; modulates gut microbiota composition	Preclinical (animal)	Time-consuming; individual variability; contraindications	(Wang et al., 2026)
Natural product intervention	Puerarin	Reduces *Prevotella copri* abundance and its TMA production → lowers plasma TMAO	Clinical (RCT, carotid plaque)	Mechanism-specific; no heat stroke trials	(Li et al., 2024b)
Antibiotic modulation	Antibiotics (experimental)	Microbiota depletion → NOX2 and TMAO inhibition → attenuates AKI–CKD transition	Preclinical (animal)	Dysbiosis risk; antimicrobial resistance	(Lee et al., 2024)

## 8 Conclusions and future perspectives

TMAO, as a key gut microbiota-derived metabolite, is proposed to play multi-dimensional and multi-level roles in heat stroke pathogenesis, although direct evidence from heat-stroke models is currently lacking. The pathogenic cascade — heat stress → gut barrier disruption → gut dysbiosis → TMAO overproduction and proposed systemic release → NLRP3 inflammasome activation and inflammatory cascade → endothelial dysfunction → platelet hyperreactivity and thrombosis → mitochondrial dysfunction and bioenergetic failure → multi-organ injury — constitutes a plausible and potentially self-amplifying pathological network. However, most individual links in this network are supported by indirect evidence from non-heat-stroke models, and the network as a whole should be regarded as a working hypothesis requiring validation in heat-stroke-specific experimental and clinical studies.

Despite significant advances, several challenges remain: (1) the specific molecular receptor for TMAO in eukaryotic cells has not yet been identified, limiting mechanistic understanding of its direct molecular actions (Zhu et al., 2016); (2) the dynamic changes in TMAO concentrations across different disease stages and tissues, and their relationship with disease severity, require further clinical validation; (3) the efficacy and safety of TMAO-targeting therapeutic strategies in heat stroke models need systematic evaluation.

Future research should focus on: (1) elucidating the concentration-dependent functional switch of TMAO in heat stroke; (2) exploring the regulatory effects of non-pharmacological interventions such as heat acclimatization on TMAO levels; (3) developing gut microbiota-based novel strategies for heat stroke prevention and treatment. TMAO may hold promise as a candidate diagnostic biomarker and therapeutic target; however, clinical validation studies in heat-stroke patients and heat-stroke-specific intervention data are prerequisites before either application can be considered for translation.

### 8.1 Limitations

This narrative review has several limitations that should be acknowledged. First, the literature search was not conducted according to a systematic review protocol (e.g., PRISMA), which may have introduced selection bias. Second, most mechanistic evidence regarding TMAO derives from *in vitro* cell culture experiments and animal models, and the translational relevance to human heat stroke pathophysiology requires further investigation. Third, direct clinical studies examining TMAO levels in heat stroke patients remain scarce, limiting the clinical applicability of the proposed mechanisms. Fourth, the heterogeneity of experimental models (varying TMAO concentrations, cell types, and heat stress protocols) precludes quantitative meta-analysis. Fifth, although we attempted to identify all relevant literature, some publications may have been missed due to search limitations. Future systematic reviews with meta-analysis, when sufficient primary data become available, would strengthen the evidence base. Sixth, direct evidence obtained in heat-stroke models is scarce: many mechanistic claims rely on extrapolation from other disease settings; sample matrices and experimental readouts are inconsistent across studies; the kinetic profile of circulating TMAO during and after heat stroke is undefined; and interventional studies targeting the TMAO pathway under hyperthermic conditions are essentially absent. In particular, whether increased TMAO is a cause or a consequence of severe heat stroke—given the confounding effect of heat-induced acute kidney injury on TMAO clearance—remains unresolved (see Section 5.6). Most TMAO-related causal mechanisms in heat stroke therefore remain to be validated by original experimental work.
